# Phase I/II trial evaluating concurrent carbon-ion radiotherapy plus chemotherapy for salvage treatment of locally recurrent nasopharyngeal carcinoma

**DOI:** 10.1186/s40880-016-0164-5

**Published:** 2016-12-22

**Authors:** Lin Kong, Jing Gao, Jiyi Hu, Weixu Hu, Xiyin Guan, Rong Lu, Jiade J. Lu

**Affiliations:** 1 Department of Radiation Oncology, Shanghai Proton and Heavy Ion Center, Fudan University Shanghai Cancer Hospital, Shanghai, 200321 P. R. China; 2 Department of Radiation Oncology, Shanghai Proton and Heavy Ion Center, Shanghai, 200321 P. R. China; 3 Department of Outpatient Clinic, Shanghai Proton and Heavy Ion Center, Shanghai, 200321 P. R. China; 4 Shanghai Proton and Heavy Ion Center, 4365 Kangxin Road, Pudong, Shanghai, 201315 P. R. China

**Keywords:** Recurrent nasopharyngeal cancer, Carbon ion radiotherapy, Re-irradiation, Salvage therapy, Chemotherapy

## Abstract

**Background:**

After definitive chemoradiotherapy for non-metastatic nasopharyngeal carcinoma (NPC), more than 10% of patients will experience a local recurrence. Salvage treatments present significant challenges for locally recurrent NPC. Surgery, stereotactic ablative body radiotherapy, and brachytherapy have been used to treat locally recurrent NPC. However, only patients with small-volume tumors can benefit from these treatments. Re-irradiation with X-ray—based intensity-modulated radiotherapy (IMXT) has been more widely used for salvage treatment of locally recurrent NPC with a large tumor burden, but over-irradiation to the surrounding normal tissues has been shown to cause frequent and severe toxicities. Furthermore, locally recurrent NPC represents a clinical entity that is more radio-resistant than its primary counterpart. Due to the inherent physical advantages of heavy-particle therapy, precise dose delivery to the target volume(s), without exposing the surrounding organs at risk to extra doses, is highly feasible with carbon-ion radiotherapy (CIRT). In addition, CIRT is a high linear energy transfer (LET) radiation and provides an increased relative biological effectiveness compared with photon and proton radiotherapy. Our prior work showed that CIRT alone to 57.5 GyE (gray equivalent), at 2.5 GyE per daily fraction, was well tolerated in patients who were previously treated for NPC with a definitive dose of IMXT. The short-term response rates at 3–6 months were also acceptable. However, no patients were treated with concurrent chemotherapy. Whether the addition of concurrent chemotherapy to CIRT can benefit locally recurrent NPC patients over CIRT alone has never been addressed. It is possible that the benefits of high-LET CIRT may make radiosensitizing chemotherapy unnecessary. We therefore implemented a phase I/II clinical trial to address these questions and present our methodology and results.

**Methods and design:**

The maximal tolerated dose (MTD) of re-treatment using raster-scanning CIRT plus concurrent cisplatin will be determined in the phase I, dose-escalating stage of this study. CIRT dose escalation from 52.5 to 65 GyE (2.5 GyE × 21–26 fractions) will be delivered, with the primary endpoints being acute and subacute toxicities. Efficacy in terms of overall survival (OS) and local progression-free survival of patients after concurrent chemotherapy plus CIRT at the determined MTD will then be studied in the phase II stage of the trial. We hypothesize that CIRT plus chemotherapy can improve the 2-year OS rate from the historical 50% to at least 70%.

**Conclusions:**

Re-treatment of locally recurrent NPC using photon radiation techniques, including IMXT, provides moderate efficacy but causes potentially severe toxicities. Improved outcomes in terms of efficacy and toxicity profile are expected with CIRT plus chemotherapy. However, the MTD of CIRT used concurrently with cisplatin-based chemotherapy for locally recurrent NPC remains to be determined. In addition, whether the addition of chemotherapy to CIRT is needed remains unknown. These questions will be evaluated in the dose-escalating phase I and randomized phase II trials.

## Background

Despite substantial improvements in treatment outcomes for nasopharyngeal carcinoma (NPC) owing to new radiation technologies, such as X-ray—based intensity-modulated radiotherapy (IMXT), and concurrent chemoradiotherapy, local recurrence remains a common reason for treatment failure. Unfortunately, approximately 10%–15% of patients with non-metastatic disease will experience local recurrence after high-dose IMXT, with or without chemotherapy [1]. Re-treatment with definitive intent after high-dose IMXT poses a significant challenge to oncologists who specialize in head and neck cancer.

Several modalities have been used with some success in the re-treatment of local NPC when the initial treatments failed. For example, nasopharyngectomy, for a selected group of NPC patients with limited tumor volume in the post-nasal space, might achieve a long-term control rate of over 50% [2]. Re-irradiation using stereotactic radiosurgery or brachytherapy can also be used for recurrent foci with limited tumor volume [3, 4]. However, for more extensive local recurrences after high-dose radiation, IMXT is currently the treatment of choice in the endemic region. Several retrospective studies have addressed the long-term results of using IMXT for locally recurrent NPC; the documented 3- to 5-year overall survival (OS) rates for most patients who presented with locally advanced recurrent disease were usually less than 40% [5–7].

### Challenges in the re-treatment of locally recurrent NPC

Re-irradiation of locally recurrent NPC after a prior course of definitive radiation is challenging for many reasons. The high-dose volumes encompassed in the initial course of definitive radiotherapy for the primary disease consist of not only the gross tumor volume (GTV) but also the organs at risk (OARs), such as the pharyngeal mucosa, optic nerve/chiasm, temporal lobes of the brain, and brain stem, as necessitated for the coverage of subclinical disease. A substantial portion of the temporal lobes and most parts of the nasopharynx are often irradiated with a dose of 66–70 Gy, especially in patients with locally advanced recurrences. When the combined radiation dose of both initial radiotherapy and re-irradiation exceeds 100 Gy, the likelihood of radiation-induced adverse effects increases substantially (from 4% to 39%) [8]. Therefore, re-irradiation to a dose that exceeds 60 Gy may cause severe long-term adverse effects, including temporal lobe necrosis and soft-tissue ulceration and necrosis. Nasopharyngeal mucosal ulceration and necrosis is a potentially life-threatening condition. In addition to causing a significant reduction in quality of life, high-dose re-irradiation can cause death from necrosis, infection, or massive hemorrhage of the internal carotid artery, despite optimum clinical management.

Local recurrence of NPC after definitive radiotherapy with photons is most likely caused by radio-resistant NPC cells that survived the first course of radiation. Treatments might also fail locally due to the technical limitations of two-dimensional conventional radiotherapy or three-dimensional conformal radiotherapy, in which inadequate imaging, targeting, and delivery reproducibility may result in under-dosing of the primary disease and neck adenopathy. The prevailing use of IMXT, along with advanced imaging technology such as magnetic resonance imaging (MRI) and positron emission tomography/computed tomography (PET/CT), have significantly improved locoregional disease control over the years. Despite these advances, 10%–15% of patients who complete IMXT at 70 Gy still experience local treatment failure [1]. Therefore, having largely eliminated the technological limitations of treatment, it is reasonable to speculate that there are subgroups of cells within the gross disease that are more resistant to X-ray—based radiation and are enriched within recurrent tumors. Whether such features of radio-resistance are secondary to inherently radio-resistant clones, the presence of cancer stem cells, or hypo-oxygenation remains unknown. Regardless of the mechanisms, radio-resistance of the recurrent tumor confers a worse outcome after re-treatment using IMXT with doses similar to the initial radiotherapy (i.e., 60–70 Gy at standard fractionation). Thus, previous IMXT for primary NPC poses additional biologic challenges to re-treatment of local recurrence. This clinical scenario requires the targeting of a more radio-resistant disease while several critical OARs may have already been encompassed in the high-dose region. Nearly all patients reported in the literature so far were treated with two-dimensional conventional radiotherapy. As such, previous data may not be comparable to studies conducted in the IMXT era.

### Characteristics of particle radiotherapy in cancer treatment

Charged-particle therapy, such as proton therapy, helium therapy, and carbon-ion radiotherapy (CIRT), possess distinct physical characteristics that make it superior to photon therapy. These include a sharp lateral penumbra; minimal energy deposition within the beam’s entry path prior to the Bragg peak, which is defined by its steep dose deposition; and a sharp dose-deposition fall-off after the Bragg peak. Thus, beams exhibit both a precise and a finite range with respect to their dose delivery capability. The depth of the Bragg peak can be altered by altering the beam’s energy. These properties enable the sparing of surrounding normal tissues, which is crucial when irradiating the head and neck area, especially for patients who have received prior radiotherapy to this region. Several reports have shown improved dose distributions using particle therapy for primary or recurrent NPC, with acceptable efficacy and toxicity profiles [9–11].

In addition to its superior dosimetric properties, the heavy carbon ion is a high linear energy transfer (LET) modality. Furthermore, the relative biological effectiveness (RBE) of CIRT is substantially higher than that of photon- and even proton-based radiation. The value of RBE is 3–5 for carbon ion. The actual calculated value is dependent on both the tissue type and the biologic endpoint of RBE assessment. High-LET radiation inflicts more damage via direct DNA double-strand breaks, which are more difficult to repair [12]. Improved efficacy could be expected after the delivery of high-LET radiation, such as CIRT, especially in the treatment of photon-resistant cancer cells that have been selected for their ability to more efficiently repair DNA single-strand breaks. Per convention with CIRT (and other particle-based modalities), the differences in RBE and LET between CIRT and photon radiotherapy will be taken into account, and the CIRT doses will be reported in terms of gray equivalents (GyE), which refer to the biologic effective doses (BEDs) of photons.

Results from retrospective and prospective studies have shown improved outcomes after CIRT for several malignancies, including chordoma/chondrosarcoma of the skull base [13, 14], melanoma [15], and adenoid cystic carcinoma of the head and neck region [16]. These results also demonstrated the safety of CIRT to critical OARs such as the optical nerve/chiasm, brain, brainstem, and spinal cord. At the Heidelberg Ion-Beam Therapy Center in Germany and the National Institute of Radiological Sciences in Japan, CIRT is currently the routine treatment for patients with these conditions.

### Particle therapy for local recurrence in head and neck cancers

Particle therapy has also been used successfully in the re-treatment of local recurrences of the above-mentioned conditions of the head and neck, including locally recurrent NPC. In one study, 16 NPC patients who developed a local recurrence after conventional photon-based radiotherapy and were re-irradiated by proton radiation achieved an OS rate of 50% [11]. Those who were re-irradiated with 61 Gy or more achieved a local control rate of 60%; however, the local control rate was only 38% for those irradiated with a lower total dose (*P* = 0.17) [11]. The median cumulative dose of both the initial radiotherapy and re-irradiation was 133.5 Gy (range, 110–148 Gy). The 2 of 9 (22.2%) surviving patients who developed osteonecrosis and mucosal necrosis were successfully treated with surgical debridement and hyperbaric oxygen. With radiation doses of 60.0, 60.0, and 57.0 Gy to the optic chiasm, surface of the brainstem, and center of the brainstem, respectively, no patient experienced clinically detectable neurological dysfunction [11].

A series from the Heidelberg Ion-Beam Therapy Center included 18 patients with base-of-skull tumors who developed local recurrences after an initial course of radiotherapy and were re-irradiated with CIRT to a median dose of 51 GyE (range, 42–60 GyE) over 17 consecutive days (3 GyE per daily fraction) [17]. In this study, 3 of 18 patients received two courses of radiotherapy prior to the re-irradiation and received CIRT for their third course. Based on this calculation, the cumulative biological effective dose with an α/β ratio of 2 (BED2; usually for late-responding tissue) and cumulative biological effective dose with an α/β ratio of 9 (BED9; usually for tumor and early-responding tissue) were 127.5 and 68 GyE, respectively, using an α/β value of 2 for late toxicity of normal tissue. No patient developed grade 3 or 4 early or late toxicities.

### Concurrent chemotherapy with CIRT for locally recurrent NPC

Concurrent chemotherapy with IMXT is the current standard treatment for locally advanced NPC and has been studied against other chemotherapy scheduling [18, 19]. However, the addition of chemotherapy during IMXT for re-irradiation of locally recurrent NPC has never been studied in any prospective clinical trial. In the above-mentioned retrospective studies, some patients with recurrent NPC were treated with IMXT plus chemotherapy (either concurrent or adjuvant). However, multivariate analyses did not confirm that the addition of chemotherapy was a significant prognostic factor. The underlying mechanism of chemotherapy used with radiotherapy for recurrent NPC is not clear. However, in vitro studies have indicated the chemo-resistant characteristics of NPC stem cells [20]. It is reasonable to presume that locally recurrent NPC foci harbor chemo-resistant (and radio-resistant) cells that may reduce the efficacy of chemotherapy. In addition, the early onset of life-threatening adverse effects may mask the synergistic effects of combined chemoradiotherapy on disease control and survival.

The combination of CIRT with concurrent chemotherapy has never been studied prospectively for any malignancy. Given the superior physical and biological properties of CIRT and the potential benefits of concurrent chemotherapy, it is also reasonable to postulate that CIRT plus concurrent cisplatin-based chemotherapy may produce an optimal outcome in the treatment of locally recurrent NPC after high-dose IMXT. In this trial, we will first determine the maximal tolerated dose (MTD) of CIRT when used with concurrent cisplatin (weekly dose, 40 mg/m^2^) for the re-irradiation of locally recurrent NPC after IMXT. We will then evaluate the effectiveness of this strategy at the MTD.

### Selection of CIRT dose for re-irradiation in the phase I study

In the phase I part, we will use a Time-to-Event Continual Reassessment Method (TITE-CRM) design to determine, via a dose-escalation scheme, the MTD for re-irradiation using CIRT plus concurrent chemotherapy for the treatment of locally recurrent NPC [21, 22]. Patients will be monitored and evaluated for acute and subacute treatment-induced adverse effects, local control, local and distant progression-free survival, and OS.

Doses between 60 and 70 Gy (1.8/2.0 Gy per daily fraction) were commonly used for re-irradiation with IMXT for the treatment of locally recurrent NPC. Unfortunately, this dose range is associated with at least 35% of severe adverse effects (defined as grades 3–5 toxicities), including soft-tissue ulceration and hemorrhage, especially at higher doses [5, 7]. In a series of 239 patients treated with a mean dose of 70.04 Gy (range, 61.73–77.54 Gy) to the GTV, grades 3–5 re-irradiation-induced severe late toxicities occurred in approximately 52% of the patients and were the cause of death of almost 70% of the 120 patients who died [7]. More recently, in a study of salvage IMXT for the treatment of locally recurrent NPC after previous definitive IMXT, Kong et al. [23] reported a similar rate of severe toxicity (approximately 40%) after re-irradiation using IMXT. In addition, many patients developed two or more late toxicities after retreatment, and almost 70% of mucosal necrosis occurred within 6 months after re-treatment. Currently, a reduced dose range from 60 to 66 Gy at conventional fractionation (i.e., BED9 73–81 GyE) is commonly used for re-irradiation with IMXT for the treatment of locally recurrent NPC. However, long-term results after treatment at this dose level, especially for patients who recurred after receiving definitive IMXT for their primary treatment, have not been reported.

Two articles on re-irradiation using proton or heavy-particle therapy have reported clinically acceptable long-term toxicity outcomes. In these studies, a BED2 of approximately 120 GyE was delivered to the recurrent tumor. Doses of re-irradiation using proton and carbon ion exceeding or below 120 GyE conferred grade 3/4 late toxicity rates of approximately 22% and 0%, respectively [11, 17]. A total dose of 55 GyE (at 2.5 GyE per daily fraction) resulted in a BED2 of 124 GyE and a BED9 of 70 GyE. Since concurrent chemotherapy with CIRT may provide additive, if not synergistic, effects on efficacy as well as toxicity, 55 GyE at 2.5 GyE/day should serve as an acceptable starting dose/fraction schedule. In our previous phase I/II trial on CIRT alone for the treatment of locally recurrent NPC, we chose 57.5 GyE as the starting dose [24]. Owing to the potential additive toxicity of concurrent chemotherapy combined with CIRT, it will be prudent to set the starting dose of the phase I trial somewhat lower, at 55 GyE delivered at 2.5 GyE/day.

Because of the sharper penumbra offered by CIRT, we anticipate that the delivery of higher doses to the clinical targets—while potentially lowering the integral dose to, and high-dose volumes within, the non-targeted structures—will be more feasible compared with traditional photon-based radiotherapy. Because of these unique properties of CIRT, in addition to determining the straightforward MTD, toxicity will be associated with other unique dosimetric characteristics, such as dose-volume histogram parameters and gradient and conformality indices of the various BEDs within normal structures and tissues. This will provide supplemental three-dimensional and dynamic information to determine the tolerance of previously treated tissues to CIRT. Lastly, we will be able to determine the additive or synergistic effects of adding platinum-based chemotherapy to CIRT, as previous studies, with toxicity data, have consisted primarily of CIRT-alone regimens.

## Methods and design

As a single-center, single-arm phase I/II clinical trial, its purposes are to determine the MTD of re-irradiation using CIRT combined with concurrent cisplatin-based chemotherapy in the treatment of locally recurrent NPC and to evaluate the efficacy of this strategy at the determined MTD.

### Objectives

#### Phase I trial

The primary objective is to determine the MTD of CIRT combined with concurrent chemotherapy in the treatment of locally recurrent NPC, by observing the acute or subacute grade 4 or 5 toxicities [Common Terminology Criteria for Adverse Events (CTCAE) v. 4.03] that are possibly, probably, or definitively caused by the treatments [25]. The secondary objective is to evaluate the local progression-free survival and OS after re-irradiation using the combined treatment.

#### Phase II trial

The primary endpoint is 2-year OS after salvage CIRT with concurrent chemotherapy. The secondary endpoints are local control, disease-free survival, and long-term toxicity and safety.

### Trial design and schedule

#### Phase I trial: dose escalation to determine MTD

The purpose of the phase I trial is to determine the MTD of salvage raster-scanning CIRT with concurrent cisplatin-based chemotherapy for locally recurrent NPC. Patients who meet the inclusion criteria will be treated with chemotherapy and this dose-escalating CIRT scheme, and acute and subacute toxicity during the treatments and within 4 months after CIRT will be observed.

The study includes 6 increasing dose regimens starting at 52.5 GyE (2.5 GyE × 19 fractions) up to 65 GyE (2.5 GyE × 26 fractions) using the TITE-CRM design of phase I trials, with the starting dose/fraction at 55 GyE delivered daily in 22 fractions. The trial will be concluded at 65 GyE if the MTD is not reached, and 65 GyE/26 fractions will be the recommended dose/fractions for the phase II trial. The probability of dose limiting toxicity (pDLT) for each dose level is estimated based on published data for re-irradiation with CIRT to the head and neck area (Table 1).

**Table 1:** Treatment schedule (CIRT plus concurrent chemotherapy) for dose escalation in patients with locally recurrent nasopharyngeal carcinoma (NPC)

Dose level	Dose and fractionation	Total dose (GyE)	pDLT (%)	BED2 (GyE)	BED9 (GyE)
1	2.5 GyE × 21 fractions	52.5	<5	118.1	67.1
*2*	*2.5 GyE* *×* *22 fractions (starting dose)*	*55*	*10*	*123.8*	*70.3*
3	2.5 GyE × 23 fractions	57.5	20	129.4	73.5
4	2.5 GyE × 24 fractions	60	30	135.0	76.7
5	2.5 GyE × 25 fractions	62.5	40	140.6	79.9
6	2.5 GyE × 26 fractions	65	50	146.3	83.1

The starting dose of the trial is at 2.5 GyE *×* 22 (in italics)

*CIRT* carbon-ion radiotherapy, *GyE* gray equivalent, *pDLT* probability of dose-limiting toxicities, *BED* biological effective dose, *BED2* cumulative BED with an α/β ratio of 2, *BED9* cumulative BED with an α/β ratio of 9

A maximum of 25 patients for the 6 dose levels (starting at 55 GyE) is projected for phase I trial (i.e., the dose-escalating stage of the trial). Based on our clinical load, we expect to recruit an average of 1 patient per week. We expect that the phase I trial will be completed in 24 months, since the first patient at each dose level will be followed up for 4 months before dose escalation.

#### Phase II trial: treatment at recommended dose to study efficacy

Once the MTD is determined in the phase I trial, this dose will be used as the recommended dose for the phase II trial. If MTD is not reached at 65 GyE, we will use 65 GyE as the recommended dose.

The primary endpoint of the phase II trial is OS after re-irradiation, at a median follow-up of 24 months. To evaluate the OS rate, all patients will be followed up for at least 12 months or until death. We estimate that 1 patient per week will be recruited to the trial, and a total of 40 patients (minus the number of patients treated at the recommended dose in the phase I trial) will be recruited over approximately 9 months. The phase II trial is expected to be completed in 24 months.

### Inclusion and exclusion criteria

For this trial, all patients with a confirmed diagnosis of locally recurrent NPC will be evaluated and screened. Inclusion and exclusion criteria are listed in Table 2.

**Table 2:** Inclusion and exclusion criteria for the phase I/II CIRT plus chemotherapy dose escalation trial for NPC

Inclusion criteria	Exclusion criteria
Pathologically and/or clinically confirmed locally recurrent NPC diagnosed more than 12 months after completion of initial IMXT Completed a definitive course of IMXT to a total dose of ≥66 Gy Age ≥18 and <70 years KPS score ≥70 Ability to understand the trial protocol, including the aims, methods, and consequences Willingness to provide written informed consent before enrollment in the trial Willingness to receive the study treatment, including CIRT and cisplatin-based concurrent chemotherapy For women with childbearing potential, willingness to use adequate contraception	Presence of distant metastasis Recurrence diagnosed within 12 months after the completion of the previous IMXT course Technology used other than IMXT (or IMXT used with other techniques, such as brachytherapy or SRS) for the initial treatment Pregnant or lactating women Patients who have not recovered from severe toxicities of prior radiotherapy or chemotherapy Diagnosis of any type of cancer other than CIS of the cervix and basal cell or squamous cell carcinoma of the skin within the past 5 years Participation in other clinical trial(s) whose treatments may interfere with the conduct or outcome of this trial

*CIRT* carbon-ion radiotherapy, *IMXT* X-ray–based intensity-modulated radiotherapy, *KPS* Karnofsky performance status, *SRS* stereotactic radiosurgery, *CIS* carcinoma in situ

### Induction, concurrent, and adjuvant treatments

#### Induction and adjuvant chemotherapy

Induction chemotherapy (with or without targeted therapy) will be administered to patients with rT3, rT4, or rN+ disease (excluding those with retroperitoneal lymph node recurrence only) unless clinically contraindicated. Induction chemotherapy is not mandatory for patients with rT1, rT2, or recurrent retropharyngeal node disease only. Routine adjuvant chemotherapy is not allowed. Patients with local recurrence/progression or distant metastasis after re-irradiation with CIRT can be considered for salvage chemotherapy.

#### Concurrent chemotherapy

The administration of concurrent cisplatin-based chemotherapy will start on the first day of CIRT and will consist of weekly cisplatin (40 mg/m^2^) during CIRT for a maximum of 5 cycles. Chemotherapy at the full dose will be delivered, and no adjustment will be allowed unless grade 4 chemotherapy-induced toxicities occur. The start of chemotherapy can be postponed if a patient’s neutrophil count drops below 2000/µL or the platelet count drops below 100,000/µL. Chemotherapy can be suspended if a patient’s creatinine clearance rate is less than 50 mL/min.

#### Other medications

Prescription medication required for the treatment of medical conditions other than NPC is allowed during the phase II trial. However, any treatments not related to the trial must be discussed with and thoroughly reviewed by the principal investigator prior to a patient’s inclusion.

### Radiation therapy^1^

#### Treatment planning

Patients will be immobilized using an individual immobilization system for planning and treatment. Planning CT without contrast will be performed; MRI scans in the treatment position will be obtained, registered, and fused with a planning CT.

- GTV is defined as the gross disease seen on the planning CT, area of contrast enhancement on T1-weighted MRI, and/or lesion(s) with high standardized uptake value observed on fluoro-d-glucose-PET/CT (optional).
- Clinical target volume (CTV) is defined as the GTV plus a 3 to 5-mm margin. The CTV for subclinical disease will be delineated based on the treating physician’s clinical judgment for potential subclinical disease.
- Planning target volume (PTV) will be added depending on individual factors such as patient positioning reproducibility and/or beam angles chosen and will range from 3 to 6 mm.

Critical OARs, including the brain stem, optic nerve, optic chiasm, temporal lobes of the brain, and eyes, will be contoured. A dose discount to OARs from the initial radiation course will be uniformly set at 70%; thus, 30% residual doses will be used to calculate the limiting dose to the OARs. Dose limitations of OARs will be controlled according to those reported by Emami et al. [26].

CIRT planning will be performed using the Syngo treatment planning system (Siemens; Erlangen, Germany), including biologic plan optimization. BED distributions will be calculated using the generally accepted an α/β ratio of 9 for NPC and an α/β ratio of 2 for late-responding tissues (generally presented as late toxicity).

#### Dose prescription for CIRT

Re-irradiation using intensity-modulated CIRT will be delivered using the IONTRIS raster scan system (Siemens Aktiengesellschaft, Munich, Germany). Six dose levels and the estimation of pDLT of each level are detailed in Table 1. A daily dose of 2.5 GyE will be delivered up to the total dose for all levels and be given concurrently with chemotherapy (see “Chemotherapy” above). After the MTD is determined or if the treatments to 65 GyE are safely delivered, the delivered dose (or 65 GyE) will be defined as recommended dose and used as the prescribed dose in the phase II stage of the study.

Ninety-five percent of the isodose line should cover the CTV for gross tumor (i.e., GTV plus 3–5 mm), and 90% of the dose line should cover the PTV for gross disease. The CTV for other subclinical disease, if applicable, will be irradiated to 90% of the assigned dose level.

Dose specification is based on BED, because the RBE of CIRT differs significantly from photon therapy. Therefore, the unit used for dose prescription is the iso-effective dose GyE daily for 5 fractions per week at 2 Gy per fraction.

#### Treatment planning and delivery

**Fig. 1:**
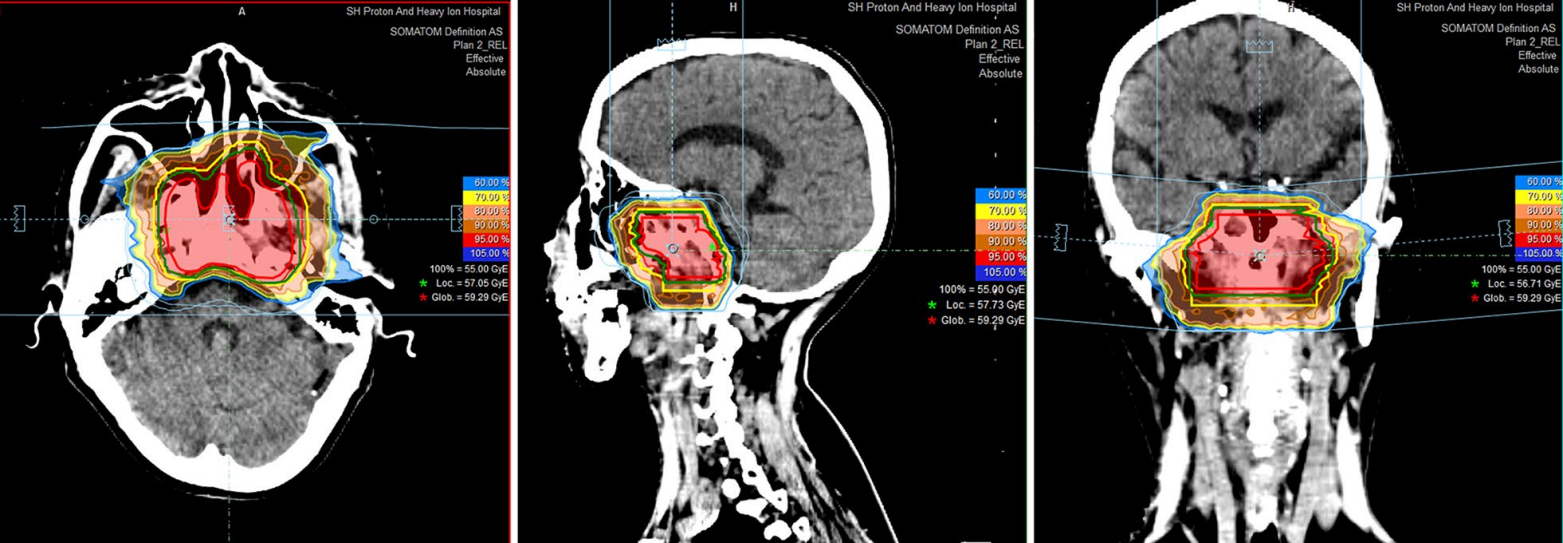
Typical treatment plan and dose distribution for intensity-modulated carbon-ion radiotherapy delivered using the raster-scanning technique in a patient with stage rT3N0M0 nasopharyngeal carcinoma with recurrence at the base of the skull. Transverse, sagittal, and coronal views are provided. The *red*, *orange*, *yellow*, and *blue shaded areas* represent 95%, 85%, 70%, and 30% isodose lines; the *red*, *green*, and *yellow* *lines* represent gross tumor volume (GTV), GTV + 3 mm, and clinical target volume (CTV), respectively

 The techniques of planning and delivery of intensity-modulated CIRT using raster-scanning technology were previously described [24]. Briefly, patients are irradiated once a day, 5 days a week. Treatment interruptions of more than 3 days are not allowed, unless severe adverse effects require such interruption. Weekend treatment will be provided if a break of 2 or more days occurred during the previous week. Treatment positioning prior to intensity-modulated CIRT will be verified by comparing orthogonal X-rays with the digitally reconstructed radiographs, and set-up deviations of more than 2 mm will be corrected. A typical treatment plan of a patient with locally recurrent NPC (rT3N0M0) is shown in Fig. 1.

### Assessment of efficacy parameters (see footnote 1)

#### Baseline documentation of “target” lesion

The target lesion is defined as the gross tumor that locally recurred in the nasopharynx or base of skull and that is scheduled for re-irradiation using CIRT. The sum of the longest diameter (LD) for the gross tumor will be measured and reported as the baseline sum LD. The baseline sum LD will be used as a reference to characterize the objective tumor.

#### Response to treatment

Response to CIRT will be recorded according to the Response Evaluation Criteria in Solid Tumors (RECIST) [26].

Evaluation of the target lesion by RECIST is summarized below.

- Complete response (CR): complete disappearance of the target lesions.
- Partial response (PR): at least a 30% decrease in the LD of the target lesion, taking the baseline sum LD as reference.
- Stable disease (SD): neither sufficient shrinkage to qualify for PR nor sufficient increase to qualify for PD, taking the smallest sum LD since the treatment started as reference.
- Progressive disease (PD): at least a 20% increase in the sum of the LD of the target lesion, taking the smallest sum LD recorded since the treatment (including neoadjuvant chemotherapy) started as reference.

Evaluation of non-target lesion(s)

- CR: disappearance of all non-target lesions and normalization of tumor marker level (not applicable for the current study).
- Incomplete response/SD: persistence of one or more non-target lesion(s) and/or maintenance of tumor marker level above the normal limits (not applicable for the current study).
- PD: appearance of one or more new lesions and/or unequivocal progression of existing non-target lesions.

Evaluation of best overall response

The best overall response will be considered the best extent of response recorded from the initiation of the study treatment until progression/recurrence of disease (taking as reference for PD with the smallest measurement of LD recorded since the treatment started). In general, the patient’s best response assignment will depend on the achievement of both measurement and confirmation criteria as follows.

- Patients will be classified as having “symptomatic deterioration” if, without disease progression, they experience an overall deterioration of general health status that necessitates discontinuation of chemoradiotherapy. Documentation of objective progression of the disease during and after the study treatment will be required.
- In some circumstances, it may be difficult to distinguish residual disease from normal tissue. When the evaluation of CR depends on this determination, it is recommended that the residual lesion be histologically investigated (by fine or core needle biopsy, for example) to confirm the CR status.

#### OS

The duration of OS will be calculated as the time between pathologic confirmation of local recurrence of NPC and the date of death from any cause. Patients who do not die or who are not lost to follow-up will be censored at the date of the last follow-up.

#### Progression-free survival

Progression-free survival will be defined as the time between the initiation of neoadjuvant chemotherapy or the start of CIRT and the date of disease recurrence or progression at any body part. Patients who do not die or who are not lost to follow-up and present no evidence of disease progression will be censored at the date of the last follow-up.

### Assessment of toxicity, safety, and other parameters

#### Toxicity and safety parameters

CTCAE v. 4.03 will be used for the monitoring and reporting of all toxicity and adverse events observed during and after the completion of study treatments [25]. Patients will be evaluated weekly by complete history, physical examination, and lab tests to determine the safety and toxicity of induction chemotherapy (if applicable) and CIRT plus concurrent chemotherapy. These evaluations will be conducted at each on-treatment appointment and at each follow-up clinical visit.

#### Other parameters

Peripheral blood will be collected to measure circulating Epstein-Barr virus (EBV) DNA and/or other tumor markers. These parameters may be used to predict the extent of disease or treatment outcome, or for future research purposes that may or may not be directly related to the current trial. A separate informed consent for this specimen collection will be required from the patients.

### Follow-up after trial completion

#### Follow-up

After completion of the study treatment, all patients will be required to be followed up regularly, indefinitely or until death, according to our institutional follow-up protocol for head and neck cancers. The first and second follow-up will be scheduled at 1 and 3 months after the completion of radiotherapy, respectively. Unless otherwise clinically necessary, follow-up sessions will then be scheduled every 3 months in the first 3 years, every 6 months in the following 2 years, and annually thereafter. Each follow-up examination will include a complete history and physical examination, MRI or CT scans with contrast of those for the head and neck area, and blood tests (including complete blood counts, serum electrolyte levels, pituitary function, and liver/renal function), and EBV DNA copies. Whole-body PET/CT scans is optional but preferable over thoracic and/or abdominal CT scans, and bone scans if clinically indicated.

#### Treatment at tumor progression

After completion of CIRT plus concurrent chemotherapy, further treatment, including salvage chemotherapy, surgical resection, a third course of radiation for locoregional recurrence, systemic treatment with chemotherapy, or targeted therapy may be clinically necessary in case of disease recurrence or progression. These treatment decisions will be made at the discretion of the treating physician and/or team.

### Statistics (see footnote 1)

#### Phase I trial

The primary objective is to determine the CIRT dose when used with concurrent cisplatin-based chemotherapy, which is associated with a DLT in less than 25% of previously treated NPC patients with a local recurrence. DLT is defined as any grade 4 toxicity (CTCAE v. 4.03) that is possibly, probably, or definitely associated with CIRT that occurred within 6 months after completion of re-irradiation [24]. Treatment-induced adverse effects also include deterioration in Eastern Cooperative Oncology Group performance status to ≥3 that develops during the same follow-up period. The secondary objectives are OS and local progression-free survival. The MTD of CIRT for re-irradiation used with concurrent cisplatin-based chemotherapy will be determined at the end of phase I trial.

Dose levels of CIRT will be assigned based on the TITE-CRM algorithm [21]. During the trial, pDLT will be continually updated, using data from all enrolled patients and their updated outcomes obtained through follow-up visits. Patients with partial follow-up at a time of new enrollment (i.e., <4 months) will be weighted by the proportion of the follow-up time completed. Newly enrolled patients will be assigned to the dose that is estimated to have a pDLT closest to but not higher than 0.25.

Four patients will be entered to the starting dose level. Dose escalation is restricted to one level between any 2 patients. Prior to escalation, at least 1 patient must have completed the full course of treatment and the full observation period (6 months) at the previous dose level without a DLT. Six dose levels and their initial estimation of the pDLT are planned within the phase I trial (Table 1). The dose will be increased until DLT is observed or when 65 GyE is reached.

As described above, the phase I trial was designed to accrue up to 25 patients. We estimate that 4 patients will be accrued each month. Patients who complete 90% or more of the planned CIRT dose will be considered evaluable. Patients who complete 90% or more of the planned CIRT dose but become un-evaluable for 6 months will be counted as evaluable in the final analysis and weighted by the proportion of the observation period for which they were evaluable. Replacement of any accrued patient can only be considered if the patient did not complete therapy for reasons other than toxicity.

At the end of the trial, a simple two-parameter logistic regression model will be used to estimate the pDLT at each dose level. Secondary endpoints are the response of tumor to CIRT, local progression-free survival, OS, and other toxicity and safety data on the studied dose levels. The Kaplan–Meier method will be used to evaluate the OS and local progression-free survival of all patients. SAS v.9.1 software (SAS Institute Inc, Cary, NC, USA) will be used for statistical analyses. The Cox proportional hazards model will be used for multivariate analysis, as the univariate results indicate.

#### Phase II trial

The primary objective of the phase II trial is to evaluate the actuarial 2-year OS rate (π) for patients with locally recurrent NPC who receive CIRT plus concurrent cisplatin-based chemotherapy. The 2-year OS rate for patients with locally recurrent rT3 or rT4 NPC range from 40% to 50% after IMXT or proton re-irradiation [5, 7].

This is the first study to address the efficacy of re-irradiation with CIRT plus concurrent cisplatin-based chemotherapy on locally recurrent NPC. We estimate that CIRT will improve 2-year OS rate by 20% to 70%, compared with IMXT or proton therapy. Thus, confirmatory analysis of the primary endpoint assesses the following test problem: H0: π ≤ 0.50 = π0 versus H1: π > 0.70. The same single-stage design will be used for the phase II trial [27]. The null hypothesis is defined by the true OS rate being 50%, and it will be tested against a one-sided alternative. A sample size of 37 patients will be required, and the null hypothesis will be rejected if 23 or more survivors are observed among 37 patients, with a median follow-up of 2 years. This design yields a type I error rate of 0.05 and a power of 0.8 if the true OS rate is 70%.

## Data collection/safety/management and ethical/legal aspects

All clinical trials of the Shanghai Proton and Heavy Ion Center (SPHIC) are required to adhere to the policies set forth by the Institutional Academic Committee and the Institutional Review Board (IRB). The following sections were adapted and summarized from the institutional policies on human studies.

### Data safety monitoring board

The IRB of the SPHIC is the independent data safety monitoring board to monitor the recruitment, the report of adverse events, and the data quality for all clinical trials. Data and interim results for this trial will be reviewed semi-annually based on the review of the trial protocol. The IRB will provide the principal investigator with requirements and recommendations on the modification of the trial, which may or may not include termination of the trial.

### Data collection and management

The Chinese Good Clinical Practice regulation requires that all clinical trial documents be kept for at least 5 years after completion of the trial. The Health and Family Planning Commission of the Shanghai Municipal Government requires that all patient medical charts, including all imaging studies, be maintained for at least 7 years. The Research Unit and the Medical Record Unit of the Department of Medical Affairs in the SPHIC will be responsible for archiving the research documents and medical charts, respectively.

### Ethical and legal aspects

IRB approval of the current trial was obtained on October 30, 2015. The accrual of patients was initiated on November 18, 2015. The study will be conducted according to the Good Clinical Practice guidelines of China and the principles of the Declaration of Helsinki (2008 version, adopted at the 59th World Medical Association General Assembly, Seoul, Korea, October 2008).

## Discussion

The management of locally recurrent NPC after definitive IMXT poses a major clinical challenge, since the recurrent disease may be more resistant to a repeated course of IMXT or other photon-based modalities. Previous studies of re-irradiation with IMXT showed suboptimal outcomes [5–7], and it is reasonable to postulate that recurrent lesions after definitive IMXT, which are usually fully encompassed by the high-dose target volume, may harbor cells that are more resistant to photon-based irradiation. Clearly, more effective local treatment is needed.

An effective re-treatment modality should have two important simultaneous characteristics: being efficacious in disease control and being mild or moderate in causing adverse effects to the surrounding OARs. Owing to its physical properties, CIRT is a precision radiotherapy modality. Additionally, it has higher RBE compared with lower-LET irradiation; therefore, it can be more effective in the treatment of neoplasms that are more resistant to photon or proton therapy.

Compared with IMXT or proton-beam therapy, CIRT for re-irradiation has been shown to improve treatment outcomes for several diseases [28]. Previous studies of CIRT in the re-treatment of locally recurrent tumors, including those that recurred in the base of the skull, after previous courses of high-dose irradiation have indicated overall safety and effectiveness [17]. However, no patients with locally recurrent NPC were included in any of these studies. Although the RBE of CIRT for NPC cells remains to be investigated, preclinical data have demonstrated that the RBE ranges from 2 to 5 for radio-resistant cells, such as glioblastoma [29, 30] and melanoma [31]. In addition, early results from clinical studies have indicated that such advantages might translate into improved clinical outcomes against these resistant recurrences [32].

Our previous work at the SPHIC showed that patients with locally recurrent NPC who received re-irradiation alone using intensity-modulated CIRT up to 57.5 GyE in 25 fractions tolerated it well [24]. Within 3 months after completion of CIRT, most patients achieved a PR or CR, without grade 2 or higher radiation-induced adverse effects. However, longer follow-up is needed to determine the likelihood of developing late CIRT-induced toxicities. A phase I/II clinical trial with the aims of determining the MTD of re-irradiation with CIRT and the efficacy of CIRT at the MTD is ongoing [24]. Induction chemotherapy may be used for the treatment of locally advanced NPC at recurrence to reduce the tumor burden and to control subclinical distant metastases. However, chemotherapy is not being used concurrently with CIRT.

Concurrent chemotherapy with definitive radiotherapy has been proven effective in the treatment of NPC [18]; however, its effectiveness in the management of locally recurrent NPC has not been confirmed. In addition, the use of concurrent chemotherapy plus high-LET irradiation, such as CIRT, for the purpose of radiosensitization has never been addressed. Therefore, after the initiation of the previous phase I/II trial that focused on CIRT alone and after completing the accrual of patients for the first dose level (57.5 GyE with a daily fraction dose of 2.5 GyE), we initiated this trial to address the safety and effectiveness of CIRT plus concurrent cisplatin-based chemotherapy. Although all patients completed 57.5 GyE without experiencing any moderate or severe adverse effects (unpublished data), the starting dose used in the current study was 55 GyE, which is one echelon lower than the starting dose used in our CIRT-alone trial. This was done to avoid possible severe adverse effects from the potential synergistic effects of concurrent chemotherapy plus CIRT.

In summary, this trial will evaluate the safety and effectiveness of intensity-modulated CIRT (using the pencil-beam scanning technique) delivered concurrently with cisplatin-based chemotherapy to patients with locally recurrent NPC. In the phase I trial, dose escalation will be performed to determine the MTD (i.e., the recommended dose) of intensity-modulated CIRT that can be prescribed with concurrent chemotherapy for locally recurrent NPC lesions. Thereafter, the phase II trial will address the effectiveness of intensity-modulated CIRT plus concurrent cisplatin at this recommended dose as definitive treatment for locally recurrent NPC.

## Footnotes

1 The radiotherapeutic techniques, doses used and treatment goals, assessment of efficacy parameters, and statistical calculation of this clinical trial are similar/identical to those of our previous clinical trial of CIRT alone [24]. However, for clarity, practicality, and ease of reading, our methods within the context of this proposal are still described in their respective sections.
